# Decreased Bone Volume and Bone Mineral Density in the Tibial Trabecular Bone Is Associated with *Per2* Gene by 405 nm Laser Stimulation

**DOI:** 10.3390/ijms161126028

**Published:** 2015-11-16

**Authors:** Yeong-Min Yoo, Myung-Han Lee, Ji Hyung Park, Dong-Hyun Seo, Sangyeob Lee, Byungjo Jung, Han Sung Kim, Kiho Bae

**Affiliations:** 1 Department of Biomedical Engineering, Yonsei-Fraunhofer Medical Device Laboratory, Yonsei University, Wonju, Gangwon-do 26493, Korea; yyeongm@hanmail.net (Y.-M.Y.); hyungpump@hanmail.net (J.H.P.); seodongh@gmail.com (D.-H.S.); sangyeob.lee.90@yonsei.ac.kr (S.L.); bjung@yonsei.ac.kr (B.J.); hanskim@yonsei.ac.kr (H.S.K.); 2 Division of Biological Science and Technology, Yonsei University, Wonju, Gangwon-do 26493, Korea; blue-me914@hanmail.net

**Keywords:** tibia, trabecular bone, *Per2*, *ALP*, *Runx*, low-level laser therapy, minimally invasive laser needle system

## Abstract

Low-level laser therapy/treatment (LLLT) using a minimally invasive laser needle system (MILNS) might enhance bone formation and suppress bone resorption. In this study, the use of 405 nm LLLT led to decreases in bone volume and bone mineral density (BMD) of tibial trabecular bone in wild-type (WT) and *Per2* knockout (KO) mice. Bone volume and bone mineral density of tibial trabecular bone was decreased by 405 nm LLLT in *Per2* KO compared to WT mice at two and four weeks. To determine the reduction in tibial bone, mRNA expressions of alkaline phosphatase (*ALP)* and *Per2* were investigated at four weeks after 405 nm laser stimulation using MILNS. *ALP* gene expression was significantly reduced in the LLLT-stimulated right tibial bone of WT and *Per2* KO mice compared to the non-irradiated left tibia (*p* < 0.001). *Per2* mRNA expression in WT mice was significantly reduced in the LLLT-stimulated right tibial bone compared to the non-irradiated left tibia (*p* < 0.001). To identify the decrease in tibial bone mediated by the *Per2* gene, levels of runt-related transcription factor 2 (*Runx2*) and *ALP* mRNAs were determined in non-irradiated WT and *Per2* KO mice. These results demonstrated significant downregulation of *Runx2* and *ALP* mRNA levels in *Per2* KO mice (*p* < 0.001). Therefore, the reduction in tibial trabecular bone resulting from 405 nm LLLT using MILNS might be associated with *Per2* gene expression.

## 1. Introduction

Although the biological mechanisms of low-level laser irradiation or low-level laser therapy/treatment (LLLT) have not been fully revealed, LLLT is a successfully and widely used non-pharmacological method for bone regeneration in experimental *in vivo* and *in vitro* models [1,2,3,4,5,6,7,8,9,10,11,12]. Current research suggests that LLLT stimulates the proliferation and differentiation of various cell types and promotes the repair process *in vivo* and *in vitro*: a wavelength of 600–904 nm and an output power of 1–500 mW; activation of ERK1/ERK2 and a phosphatidylinositol 3-kinase (PI3K) pathway; the increase of reactive oxygen species (ROS) and ATP/cyclic AMP; the absorption of light by a photoreceptor and the photoactivation of enzymes in the mitochondria; *etc.* [1,2]. With respect to bone, LLLT has been shown to modulate inflammatory processes and bone repair [3,4,5], accelerate osteoblast proliferation and bone formation [6,7,8,9,10], and enhance bone healing [1,11,12].

Low-level laser irradiation (LLLT) can scatter across the skin surface, limiting penetration to the deep bone layers. Thus, LLLT has been directly applied to the bone site of interest through tissue incision. In addition, initial photon density and therapeutic efficacy of LLLT is reduced by light-tissue interaction, such as absorption and scattering [13,14]. LLLT (660 nm wavelength) using a MILNS has been developed to overcome these light limitations in tissue. The effectiveness of this technique for preventing trabecular bone loss has been demonstrated in previous research [13,15,16].

The circadian clock genes *Period 2* (*Per2*) and *Cryptochrome 2* (*Cry2*) regulate distinct pathways in bone volume; *Cry2* chiefly influences the osteoclasts, and *Per2* acts on osteoblasts, indicating that *Per2* and *Cry2* differentially balance bone formation [17,18]. Mammalian Cry protein with flavin adenine dinucleotide (FAD) serves as a blue-light photoreceptor of 405 nm wavelength in murine cells [19,20]. The Kushibiki group has shown that LLLT (405 nm) can promote osteogenesis and reduce adipogenesis of mouse mesenchymal stromal cells (MSCs) by inducing translocation of Cry1 and Per2 proteins and decreasing *Cry1* mRNA level. LLLT can effectively control the *in vitro* fate of MSCs as a therapeutic strategy by suppressing Cry transcription [19,20]. Therefore, the present study demonstrated that 405 nm laser stimulation using MILNS applied *in vivo* tibial trabecular bone in WT and *Per2* gene KO mice to investigate bone volume and BMD with an *in vivo* micro-CT and expressions of *Per2*, *Runx2*, and *ALP* mRNAs through real-time quantitative polymerase chain reaction.

## 2. Results

**Figure 1:**
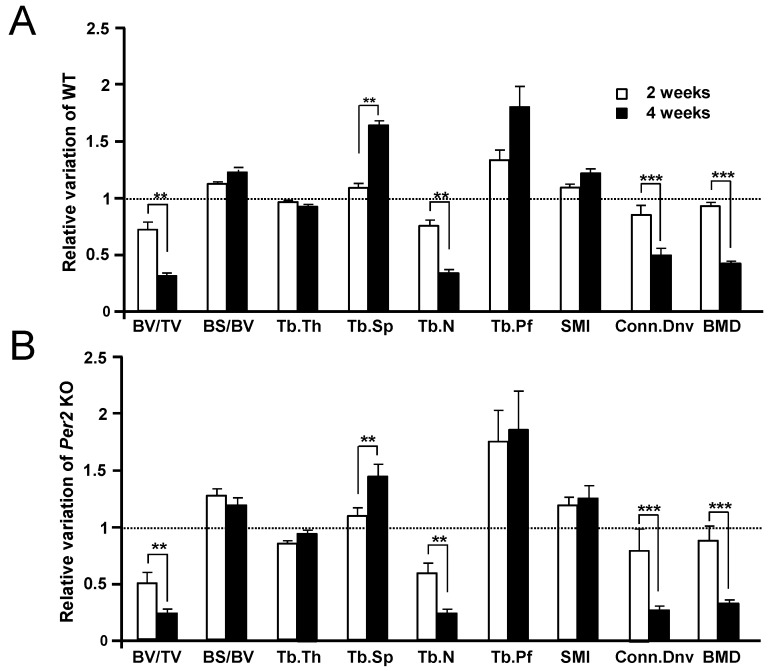
Structural parameters in 405 nm LLLT using MILNS in WT (**A**) and *Per2* KO mice (**B**), respectively. Trabecular bone parameters measured with micro-CT and depicted as a histogram. Dotted line indicates relative variation at the start of LLLT using MILNS. The tibia was directly irradiated with LLLT using the MILNS (405 nm, 5 mW, 3 J/cm^2^, 600 s). Mice were irradiated five days/week for four weeks. At two and four weeks after laser irradiation, BV/TV (%), Tb.Th (mm), Tb.Sp (mm), Tb.N (mm^−1^), Tb.Pf (mm^−1^), SMI, Conn.Dn (mm^−3^), and BMD (g/cm^3^) were measured from two-dimensional images obtained using CT-AN 1.8. Data are expressed as mean ± SEM (*n* = 9) at each point and were subjected to statistical analysis, ** *p* < 0.01; ***p < 0.001. LLLT: Low-level laser therapy/treatment; MILNS: minimally invasive laser needle system; WT: wild-type; BV/TV: structural parameters including bone volume fraction; Tb.Th: trabecular thickness; Tb.Sp: trabecular separation; Tb.N: trabeculae number; Tb.Pf: trabecular bone pattern factor; SMI: structure model index; Conn.Dn: connective density; BMD: bone mineral density.

**Figure 2:**
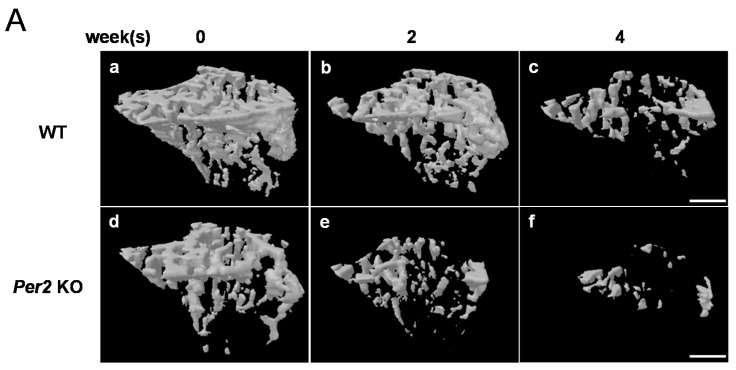

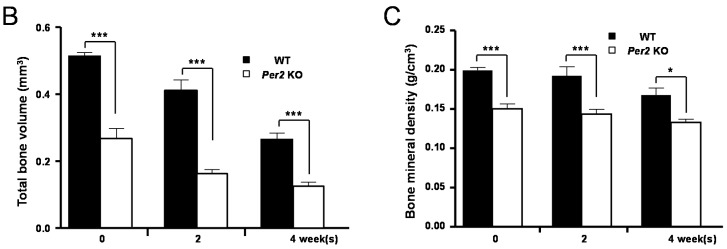
(**A**) Representative longitudinal 3D micro-CT images showing changes in right trabecular bone microarchitecture regions of interest in 405 nm LLLT using MILNS in WT and *Per2* KO mice: (**A**) **a** and **d**, 0 weeks; **b** and **e**, two weeks; and **c** and **f**, four weeks. The tibia was directly irradiated with LLLT using the MILNS (405 nm, 5 mW, 3 J/cm^2^, 600 s). Mice were irradiated five days/week for four weeks. Scale bar, 0.5 mm; At 0, two, and four weeks after laser irradiation, total bone volume (mm^3^) (**B**) and bone mineral density (g/cm^3^) (**C**) were measured on two-dimensional images obtained using CT-AN 1.8. Data are expressed as mean ± SEM (*n* = 9) and were subjected to statistical analysis, * *p* < 0.05; *** *p* < 0.001.

The structural parameters to quantify tibial trabecular bone changes using micro-CT systems were shown in the 405 nm laser-irradiated WT and *Per2* KO mice, respectively (Figure 1). The bone microarchitecture values of BV/TV, Tb.Sp, Tb.N, Conn.Dn, and BMD were significantly changed in the 405 nm laser-irradiated WT and *Per2* KO mice at four weeks compared to two weeks, respectively (BV/TV, Tb.Sp, and Tb.N, *p* < 0.01; Conn.Dn and BMD, *p* < 0.001) (Figure 1). Three-dimensional (3D) images of decreased trabecular bone were seen in *Per2* KO mice compared to WT mice that received 405 nm laser irradiation at 0, 2 and 4 weeks (Figure 2A). Changes in structural parameters were evaluated with total bone volume and BMD in WT mice and *Per2* KO mice at 0, 2 and 4 weeks (Figure 2B,C). Total bone volume and BMD were significantly reduced in *Per2* KO mice compared to WT mice (*p* < 0.001).

**Figure 3:**
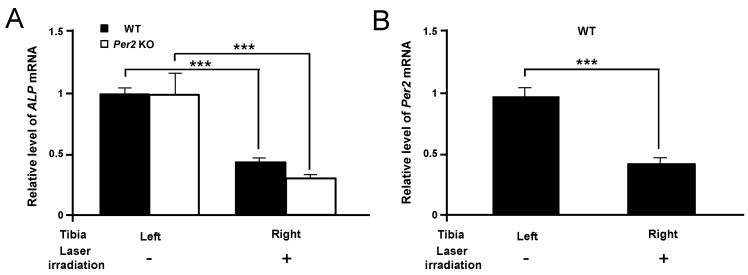
The expressions of *ALP* (**A**) and *Per2* (**B**) mRNAs in tibial bone marrows of WT and *Per2* KO mice, respectively, in 405 nm LLLT using MILNS. The data are mean ± SEM (*n* = 3). Statistical significance is indicated by *** *p* < 0.001.

To measure the reduction of tibial bone affected by LLLT using MILNS, levels of *ALP* and *Per2* mRNA were investigated in the 405 nm laser-irradiated WT and *Per2* KO mice at four weeks, respectively (Figure 3). *ALP* mRNA level was significantly reduced in right tibial bone of WT and *Per2* KO mice compared to left tibia, respectively (*p* < 0.001) (Figure 3A). In addition, *Per2* mRNA level in WT mice was significantly lower in right tibial bone compared to left tibia (*p* < 0.001) (Figure 3B).

**Figure 4:**
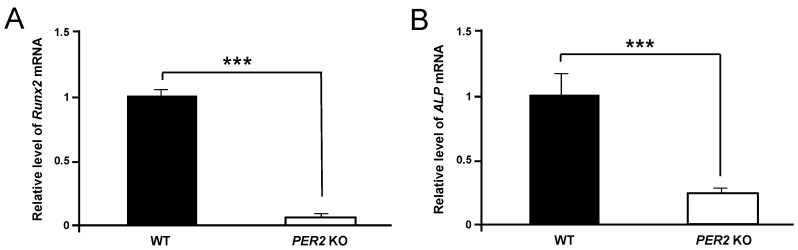

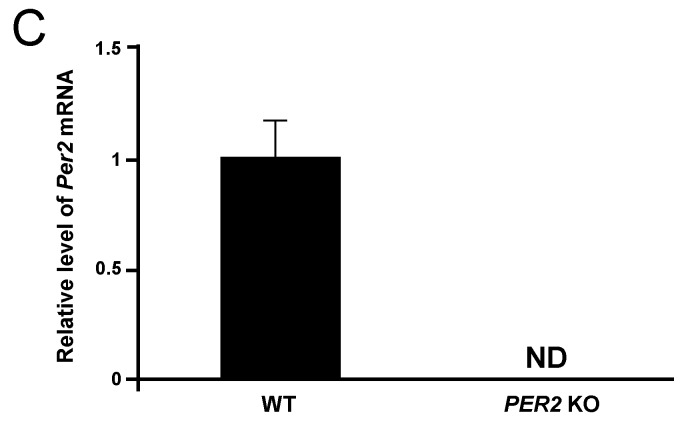
Differences in mRNA expressions of *Runx2* (**A**), *ALP* (**B**), and *Per2* (**C**) genes between tibial bone marrows of WT and *Per2* KO mice in 405 nm LLLT using MILNS. The data are mean ± SEM (*n* = 3). Statistical significant is indicated by *** *p* < 0.001. ND, no detection.

To identify the decrease in tibial trabecular bone via *Per2* gene, levels of *Runx2* and *ALP* mRNA were determined in non-irradiated WT and *Per2* KO mice, demonstrating significant down-regulation of *Runx2* and *ALP* mRNA levels in *Per2* KO mice compared to WT mice (*p* < 0.001) (Figure 4A,B). *Per2* mRNA was expressed in WT mice and was not detected in *Per2* KO mice (Figure 4C). Therefore, the reduction in tibial trabecular bone by 405 nm laser irradiation according to MILNS might be associated with *Per2* genes.

## 3. Discussion

*In vivo*, Micro-CT system is an effective and noninvasive method for the evaluation of microstructural characteristics of bone tissues. Using micro-CT allows for clear and accurate imaging of internal and external bone structures in the smallest bone fraction [21,22,23,24]. The internal and external architecture of bone has a major impact on its mechanical properties. The mechanical properties of cancellous bone are relevant to its stiffness and strength [21,22,23,24]. The biomechanical stiffness and strength of bone depend on both bone internal architecture and BMD [25]. This study was evaluated by several structural parameters including tibial trabecular bone volume fraction (BV/TV), trabecular separation (Tb.Sp), trabecular number (Tb.N), and connective density (Conn.Dn) (Figure 1) as well as total bone volume (Figure 2B) and BMD (Figure 2C). Bone densitometry using quantitative micro-CT systems provides clinical information about BMD implicated in the BV/TV. Both BMD and BV/TV can estimate the stiffness and strength of normal and pathologic trabecular bone induced by osteoporosis or metastatic cancer [26]. BMD has also been used clinically to evaluate osteoporosis and fracture risk [27]. Our findings demonstrated that total changes in total bone volume and BMD from trabecular bone of right tibia were significantly reduced in *Per2* KO mice compared to in WT mice due to 405 nm LLLT according to MILNS at 0 to four weeks (Figure 2B,C). The 3D images obtained from micro-CT clearly identified decreased trabecular bone of right tibia in *Per2* KO mice compared to in WT mice (Figure 2A). This result suggests that the reduction in tibial trabecular bone caused by 405 nm LLLT using MILNS is mediated by the *Per2* gene. Unfortunately, this study is lack of micro-CT analysis in the trabecular bone of left tibia. However, we showed that the expressions of *ALP* mRNA was significantly different between the right and left tibial bones (Figure 3A)

This study used micro-CT data to demonstrate that LLLT using MILNS led to significant reductions in tibial trabecular bone. Our results are contrary to previous studies that reported the capability of LLLT using MILNS to stimulate the cortical bone growth of osteoporotic mice, prevent trabecular bone loss in ovariectomized mice, and suppress trabecular bone loss induced by skeletal unloading [13,15,16]. These previous studies reported that LLLT using MILNS has potential to inhibit bone loss at a wavelength of 660 nm, energy of 3 J/cm^2^, output power of 10 mW, and irradiation time 300 s for five days per week for two weeks, indicating that it enhances bone formation and suppresses bone resorption. However, the present study applied a wavelength of 405 nm, energy of 3 J/cm^2^, output power of 5 mW, and irradiation time of 600 s for five days per week for two and four weeks (Figure 2B,C). The other experiment also demonstrated decreased trabecular bone loss at a wavelength of 405 nm, energy of 3 J/cm^2^, output power of 10 mW, and irradiation time 300 s (data not shown). However, the Kushibiki group used a wavelength of 405 nm, 100 mW/cm^2^, 180 s, and two weeks and demonstrated that LLLT enhanced mesenchymal stromal cell differentiation to osteoblasts *in vitro* [19,20]. Therefore, an excessively high laser dosage might lead to inhibitory effects on cell growth and proliferation [28,29,30]. Two reviews have reported that LLLT at wavelengths ranging from 600 to 904 nm and output powers of 1–500 mW is very helpful in enhancing the proliferation of various cell lines and improving bone healing [1,2]. Tajali *et al.* [3] performed a meta-analysis through MEDLINE, EMBASE, PubMed, CINAHL, and Cochrane Database of Randomized Clinical Trials published from 1966 to October 2008. Specifically, they looked for studies that highlighted the effects of LLLT on biomechanical properties of bone regeneration and the dose impact in animals. These reports might provide sufficient evidence to support the role of LLLT in animal and human bone healing.

The role of the *Per2* gene in bone is not clear, but previous studies have shown that *Per2* plays a significant role in regulating bone growth [17,18]. *Per2* KO mice demonstrate increases in bone mass and bone volume [17,18]. In children with Smith-Magenis syndrome, a genetic disorder associated with skeletal malformations, the *Per2* gene is also expressed with high variability and no *Per2* rhythm [31]. *Per2* might also influence bone growth by altering p21 cell cycle progression, through its effects on ER-mediated gene expression and its action on parathyroid hormone administration [32,33,34]. However, in the present study, *Per2* KO mice showed significantly reduced bone volume and BMD in tibial trabecular bone due to LLLT using MILNS (Figure 1 and Figure 2B,C).

Runt-related transcription factor 2 (*Runx2*) is a master transcription factor of osteoblast differentiation or osteogenesis. *Runx2* expression is upregulated in immature osteoblasts, decreases during bone development, and demonstrates a mature phenotype in osteoblasts, which are required for mature bone formation [35,36]. *ALP* plays an essential role in the bone formation process and reflects osteoblastic activity [37,38]. *ALP* is used clinically as a marker of bone formation. LLLT has a biostimulatory effect on bone formation and increases *ALP* expression or activity. Previous studies have shown that LLLT induces a significant increase in expression of *Runx2* and *ALP* mRNAs [4,39,40,41,42,43]. LLLT effects on osteoblast proliferation and bone formation involving the increase of *Runx2* mRNA [6,8]. The present study, however, demonstrated that *Per2* KO mice rather than WT mice significantly downregulated *Runx2* and *ALP* mRNA levels in tibial trabecular bone after LLLT using MILNS (Figure 4). This finding indicates that the reduction of *Runx2* and *ALP* mRNA levels in the tibial trabecular bone caused by 405 nm laser irradiation using MILNS is associated with the decrease of *Per2* gene expression.

## 4. Experimental Section

### 4.1. Experimental Animals

All procedures were performed according to a protocol approved by the Yonsei University of Animal Care Committee (YMC-141112-1). Male inbred 129/Sv WT and *mPer2* KO mice at 6 weeks of age (average weight 24.2 ± 0.8 g) (*n* = 9) were maintained in the animal facility of Yonsei University, Wonju, Korea. Environment of cage was controlled within standard conditions, temperature (23.5 ± 1 °C) and humidity (50% ± 5%). And also the mice were in controlled light (12:12 h light/dark (LD) schedule; light on at 7:00 a.m.), and were fed *ad libitum*. Mice were stabilized and synchronized with LD cycle in time-scheduled animal facility for at least 2 weeks.

### 4.2. LLLT Using MILNS

In this study, laser stimulation was performed using the MILNS technique previously developed by the Department of Biomedical Engineering and Yonsei-Fraunhofer Medical Device Lab [13,15,16]. A 130-μm-inner diameter fine needle was used to guide a 100-μm-diameter optical fiber. A diode laser (120 mW, 405 nm; No. ML320G2-11; ThorLabs, Newton, NJ, USA) was used as a light source. The optical power output from the diode laser at the end of the fine needle was set to 10 mW just before irradiation of the bone. The tibia was directly irradiated with the laser (405 nm, 5 mW) for 600 s (energy 3 J/cm^2^). The bone surface of the right tibia was directly irradiated percutaneously using MILNS at the proximal end of the tibia. The left tibia was not irradiated, but treated with needle. Mice immobilized by a customized restrainer were treated 5 days per week for 2 or 4 weeks without anesthesia.

### 4.3. Measurements of Structural Parameters

We scanned the tibiae before laser stimulation and after 2 or 4 weeks of laser stimulation with an *in vivo* micro-CT (Skyscan 1076; Bruker, Belgium, Germany) at a resolution of 18 μm^3^ under anesthesia. Anesthesia, using a combination of xylazine (0.5 mL/kg; Bayer Korea, Seoul, Korea) and zoletil (0.5 mL/kg; Virbac, Seoul, Korea), was performed during micro-CT scanning. To estimate the effects of LLLT, structural parameters of tibia were quantitatively measured. The structural parameters such as bone volume fraction (BV/TV, %), trabecular thickness (Tb.Th, mm), trabecular separation (Tb.Sp, mm), trabecular number per unit length (Tb.N, mm^−1^), trabecular bone pattern factor (Tb.Pf, mm^−1^), structure model index (SMI), connective density (Conn.Dn, mm^−3^), and bone mineral density (BMD, g/cm^3^) were analyzed on two-dimensional images obtained by CT-AN 1.8 (Bruker, Germany). We selected the region of interest (ROI) for microCT analyses, 1.8 mm in length at the distal metaphyseal secondary spongiosa (100 slices) from a point that is under 0.54 mm (30 slices) from the end of the proximal growth plate on the tibia.

### 4.4. Preparation of cDNA and Real-Time Quantitative Polymerase Chain Reaction (qPCR)

Total RNAs from tibial bone marrow were isolated from mice sacrificed at 4 weeks after LLLT. Total RNA from each sample was extracted using TRI reagent (MRC, Cincinnati, OH, USA), following the manufacturer’s instructions. Samples in 100 μL of TRI reagent were homogenized, and any contaminating genomic DNA was eliminated using the RNase-Free DNase kit (Promega, Madison, WI, USA). GoScript™ Reverse Transcription System (Promega) was used to synthesize cDNA from 2 μg of total RNA, according to the manufacturer’s protocol. The *gapdh* housekeeping gene was used as a constitutive control for normalization. The specific primer pairs used for real-time PCR are listed in Table 1. qPCR were carried out using SYBR Green reagent (Applied Biosystems, Foster City, CA, USA) and the StepOnePlus Systems (Applied Biosystems). PCR conditions included one cycle of 10 min at 95 °C followed by 40 cycles of 15 s at 95 °C, 30 s at 58 °C, and 30 s at 72 °C. The relative gene expression level was calculated using the 2^−△△*C*t^ method [44]. All samples were analyzed in triplicate and in three independent measures.

**Table 1:** Nucleotide sequences of the primer pairs used for real-time PCR.

Gene	Strand	Sequence	Accession No.
*Runx2*	Forward	5′-TAG CCA GGT TCA ACG ATC TG-3′	NM001145920.2
	Reverse	5′-TTC TGT CTG TGC CTT CTT GG-3′	
*ALP*	Forward	5′-ATA TAA CAC CAA CGC TCA GG-3′	NM007431.3
	Reverse	5′-AGG ATG GAT GTG ACC TCA TT-3′	
*Per2*	Forward	5′-TAT CGT GAA GAA CGC GGA TA-3′	NM011066.3
	Reverse	5′-AGC TGT GGA ACA CAC TGA CG-3′	
*Gapdh*	Forward	5′-GAC ATC AAG AAG GTG GTG AAG C-3′	NM008084.3
	Reverse	5′-GAA GGT GGA AGA GTG GGA GTT-3′	

### 4.5. Statistical Analysis

All the experiments were carried out at least three times. Data are presented as mean ± standard error of the mean (SEM). The significance of differences between groups was determined using Student’s *t*-test with SPSS 17.0 (SPSS Inc., Chicago, IL, USA). *p*-values less than 0.05 were considered statistically significant.

## 5. Conclusions

The results of our study illustrate that LLLT using MILNS plays a critical role in the decrease of bone volume and BMD in tibial trabecular bone in WT and *Per2* KO mice. This reduction of bone depended on significant downregulation of *Runx2* and *ALP* mRNA levels in *Per2* KO mice compared to WT mice. Future research should investigate whether specific pharmaceutical targeting of the *Per2* gene can serve as a new therapeutic avenue to treat bone loss conditions such as osteoporosis.
